# A comprehensive analysis of the microbiota composition and host driver gene mutations in colorectal cancer

**DOI:** 10.1007/s10637-022-01263-1

**Published:** 2022-06-21

**Authors:** Danping Yuan, Yong Tao, Haoyi Wang, Jiawei Wang, Yuepeng Cao, Wen Cao, Shou Pan, Zhaonan Yu

**Affiliations:** 1 Department of Colorectal Surgery, Ningbo First Hospital, Ningbo, China; 2 Hangzhou D.A. Medical Laboratory, Hangzhou, 310030 China

**Keywords:** Microbiota, 16S, Colorectal cancer, Driver gene mutation, Target therapy

## Abstract

Studies of both, microbiota and target therapy associated with gene mutations in colorectal cancer, (CRC) have attracted increasing attention. However, only a few of them analyzed the combined effects on CRC. we analyzed differences in intestinal microbiota of 44 colorectal cancer patients and 20 healthy controls (HC) using 16S rRNA gene sequencing of fecal samples. For 39 of the CRC patients, targeted Next Generation Sequencing (NGS) was carried out at formalin fixed paraffin embedded (FFPE) samples to identify somatic mutation profiles. Compared to the HC group, the microbial diversity of CRC patients was significantly lower. In the CRC group, we found a microbiome that was significantly enriched for strains of *Bifidobacterium*, *Bacteroides*, and *Megasphaera* whereas in the HC group the abundance of *Collinsella*, *Faecalibacterium*, and *Agathobacter* strains was higher. Among the mutations detected in the CRC group, the APC gene had the highest mutation rate (77%, 30/39). We found that the *KRAS* mutant type was closely associated with *Faecalibacterium*, *Roseburia*, *Megamonas*, *Lachnoclostridium,* and *Harryflintia*. Notably, Spearman correlation analysis showed that *KRAS* mutations were negatively correlated with the existence of *Bifidobacterium* and positively correlated with *Faecalibacterium*. By employing 16S rRNA gene sequencing, we identified more unique features of microbiota profiles in CRC patients. For the first time, our study showed that gene mutations could directly be linked to the microbiota composition of CRC patients. We hypothesize that the effect of a targeted colorectal cancer therapy is also closely related to the colorectal flora, however, this requires further investigation.

## Introduction

Colorectal cancer (CRC) is the third most common cancer in the world and it is one of the major causes of death and morbidity [1]. Among the causes of colorectal cancer development, the so-called driver mutations such as that of *APC*, *KRAS*, *BRAF*, *PIK3CA*, *SMAD4*, and *TP53* play significant roles. Huang et al. found that mutations of *KRAS*, *TP53*, *SMAD4,* and *BRAF* were associated with CRC metastasis and may be both potential biomarkers of metastasis as well as a therapeutic target in CRC [2].

The gut microbiota plays an important role in the development and progression of colorectal cancer. Several plausible mechanisms have been proposed for intestinal microbiota to bind to colorectal cancer cells such as inflammation, DNA damage effects, and non-DNA damage effects, all of which may be mechanically important [3]. There is increasing evidence that the gut microbiota and its products are linked to CRC. For example, it was reported that the existence of *Bacteroides* and *Bifidobacterium* species correlates positively with a high risk of CRC whereas the existence of *Lactobacillus* species and *Eubacterium aerofaciens* correlate negatively [4, 5]. According to some reports in the literature, the existence of *Clostridium nuclei*, *Streptococcus haemolyticus*, *Bacteroidetes fragilis enterotoxin*, *Enterococcus faecalis*, and *Escherichia coli* have been identified to be associated with the development of CRC [6, 7]. Also, the bacterial driver-passenger model could explain the microbial involvement in CRC development. In this model, a driver bacterium initiates CRC development by a transient colonization upon which it is replaced by a passenger bacterium displaying a competitive growth advantage in the tumor niche [8]. The candidate driver bacteria showed pre-carcinogenic characteristics such as the production of DNA damaging compounds, the disruption of tumor suppressor protein function, and induction of a host inflammatory response [9]. The identification of driver and passenger bacteria can therefore be used as classifiable biomarkers to detect high-risk groups or patients with CRC, respectively [10].

In recent years, the development of high-throughput sequencing technologies was a major step in cataloging the intestinal microbiome. More than 1,000 microbial species have been identified in the human gastrointestinal tract by analysis of the small subunit ribosomal RNA gene sequence. Most studies have focused on fecal samples to understand the composition of the gut microbiome during the development and progression of CRC. However, limited studies have assessed the association of intestinal microbial richness and biodiversity with driver gene mutations. In this study, we evaluated changes in the microbiome of CRC patients and healthy controls and explored driver gene mutations in CRC patients by comparing them to the wild-type.

## Materials and methods

### Samples

A total of 44 patients and 20 healthy controls (HC) were enrolled in this case–control study of which the patients had undergone resection of primary colorectal adenocarcinomas in the time frame between January and December 2020 in Ningbo First City Hospital. 39 of these patients were sequenced by next-generation sequencing. Inclusion criteria for the CRC group comprised the following: (a) diagnosed of colorectal cancer by colonoscopy and histopathology; (b) not suffering from diabetes, infectious diseases or having special dietary habits; (c) no drug and antibiotics uptake one month prior to surgery; (d) no preoperative chemoradiotherapy. The exclusion criteria for the experimental groups included the following: (a) long-term diarrhea; (b) gastrointestinal surgery or radiotherapy or chemotherapy prior to surgical treatment; (c) secondary tumors as identified by imaging techniques; (d) complications of other types of intestinal system diseases; (e) other infectious diseases. Individuals were not treated with antibiotics in the month prior to surgery but were administered antibiotics intravenously within a few hours of the resection. Fecal samples from CRC patients were taken the night before the surgery day. Biopsy samples from CRC tissues were taken during surgery. Pairs of fecal and tumor tissues were prospectively collected and stored at -80℃. The study was approved by the UCC Ethics Committee under the study number APC033.

### DNA Extraction from stool samples 

The total microbial genomic DNA was extracted from stool samples using the E.Z.N.A®Stool DNA Kit (D4015,Omega, Inc., USA) according to manufacturer’s instructions. The reagent which was designed to uncover DNA from trace amounts of sample has been shown to be effective for the preparation of DNA of most bacteria. Nuclear-free water was used for blank. The total DNA was eluted in 50 μL of Elution buffer and stored at -80 °C until measurement in the PCR by Dian Diagnostics, Hangzhou.

### PCR amplification and 16S rDNA sequencing

The V3-V4 region of the prokaryotic (bacterial and archaeal) small-subunit (16S) rRNA gene was amplified with primers 341F (5'-CCTACGGGNGGCWGCAG-3') and 805R (5'-GACTACHVGGGTATCTAATCC-3') [21]. The 5' ends of the primers were tagged with specific barcods per sample and sequencing universal primers. PCR amplification was performed in a total volume of 25 μL reaction mixture containing 25 ng of template DNA, 12.5 μL PCR Premix, 2.5 μL of each primer, and PCR-grade water to adjust the volume. The PCR conditions to amplify the prokaryotic 16S fragments consisted of an initial denaturation at 98 ℃ for 30 s; 32cycles of denaturation at 98 ℃ for 10 s, annealing at 54 ℃ for 30 s, and extension at 72 ℃ for 45 s; and then final extension at 72 ℃ for 10 min. The PCR products were confirmed with 2% agarose gel electrophoresis. Throughout the DNA extraction process, ultrapure water, instead of a sample solution, was used to exclude the possibility of false-positive PCR results as a negative control. The PCR products were purified by AMPure XT beads (Beckman Coulter Genomics, Danvers, MA, USA) and quantified by Qubit (Invitrogen, USA). The amplicon pools were prepared for sequencing and the size and quantity of the amplicon library were assessed on Agilent 2100 Bioanalyzer (Agilent, USA) and with the Library Quantification Kit for Illumina (Kapa Biosciences, Woburn, MA, USA), respectively. The libraries were sequenced on NovaSeq PE250 platform.

### Data analysis

Samples were sequenced on an Illumina NovaSeq platform according to the manufacturer's recommendations, provided by Dian Diagnostics, Hangzhou. Paired-end reads were assigned to samples based on their unique barcode and truncated by cutting off the barcode and primer sequence. Paired-end reads were merged using FLASH. Quality filtering on the raw reads were performed under specific filtering conditions to obtain the high-quality clean tags according to the fqtrim (v0.94). Chimeric sequences were filtered using Vsearch software (v2.3.4). After dereplication using DADA2, we obtained feature table and feature sequence. Alpha diversity and beta diversity were calculated by QIIME2, which the same number of sequences were extracted randomly through reducing the number of sequences to the minimum of some samples, and the relative abundance (X bacteria count/total count) was used in bacteria taxonomy. Alpha diversity and Beta diversity were analyzed by QIIME2 process, and pictures were drawn by R (v3.5.2).The sequence alignment of species annotation was performed by Blast, and the alignment database were SILVA and NT-16S.

### DNA Extraction from tumor tissue

According to the manufacturer's recommendations, the formalin fixed paraffin embedded (FFPE) sample genomic DNA were extracted using QIAamp® DNA FFPE Tissue Kit (product number: 56404). The purity of the extracted product was detected by Nanodrop2000, and the concentration was determined using Nanodrop2000 (Thermo) and Qubit 3.0 (Invitrogen). The extracted DNA samples were retained for subsequent experiments.

### Library preparation and next generation sequencing

Genomic DNA was randomly sheared into fragments of 150–200 bp in length by Covaris. The library of qualified genomic DNA was subjected to construct, and the sequencing libraries were generated using SureSelectXT HS Target Enrichment System. The library quality (concentration and insert size) was assessed on the Qubit 3.0 Fluorometer (Invitrogen) and Agilent Bioanalyzer 4200 system. Then, the library was diluted to 1.4 pM. Finally, targeted sequencing was carried out using the Illumina Nextseq500 platform (Illumina) and 150 bp paired-end reads were generated. This targeting panel contains 18 genes with single nucleotide variate (SNV), insertions/deletions (InDel), copy number variation (CNV), and gene fusions, including *AKT1*, *APC*, *BRAF*, *EGFR*, *ERBB2*, *FBXW7*, *HRAS*, *KRAS*, *MET*, *MLH1*, *MSH2*, *MSH6*, *NRAS*, *NTRK1*, *PIK3CA*, *PMS2*, *PTEN* and *TP53*. The average sequencing depth was > 1000X. The preparation of libraries and next generation sequencing were performed by Dian Diagnostics, Hangzhou. The SAM tools and Picard (http://broadinstitute.github.io/picard/) software were implemented to rearrange and correct the bam files to obtain the final bam file [11]. The somatic variations were detected using Mutect software [12].

## Results

### Patient characteristics

A total of 44 patients with CRC and 20 healthy controls were included in the study (Table 1). The 44 CRC patients included 20 males and 22 females, with a median age of 65 years (range 54–84 years). The medical record of all patients was retrospectively collected. The healthy controls comprised 10 males and 10 females, ranging in age from 54 to 84 years with an average of 65 years. There were no significant differences (p > 0.05) between the two groups in terms of gender, age, BMI, and other general data.

**Table 1:** Clinical features of CRC patients

Characteristics	n (Frequency)
**Gender**	
Male	20
Female	22
**Age (years)**	
Average	65 Range: 54–84
**Differentiation**	
Rectal malignancy	25
Sigmoid colon cancer	9
Ascending colon malignancy	6
Transverse colon malignancy	1
Malignant tumors of the descending colon	1
**AJCC stage**	
I	6
II	18
IIA	5
III	9
IIIB	1
IIIC	3
**Regional lymph node metastasis**	
N0	29
N1	9
N2	4

### CRC patients’ Genetic mutation information

**Fig. 1:**
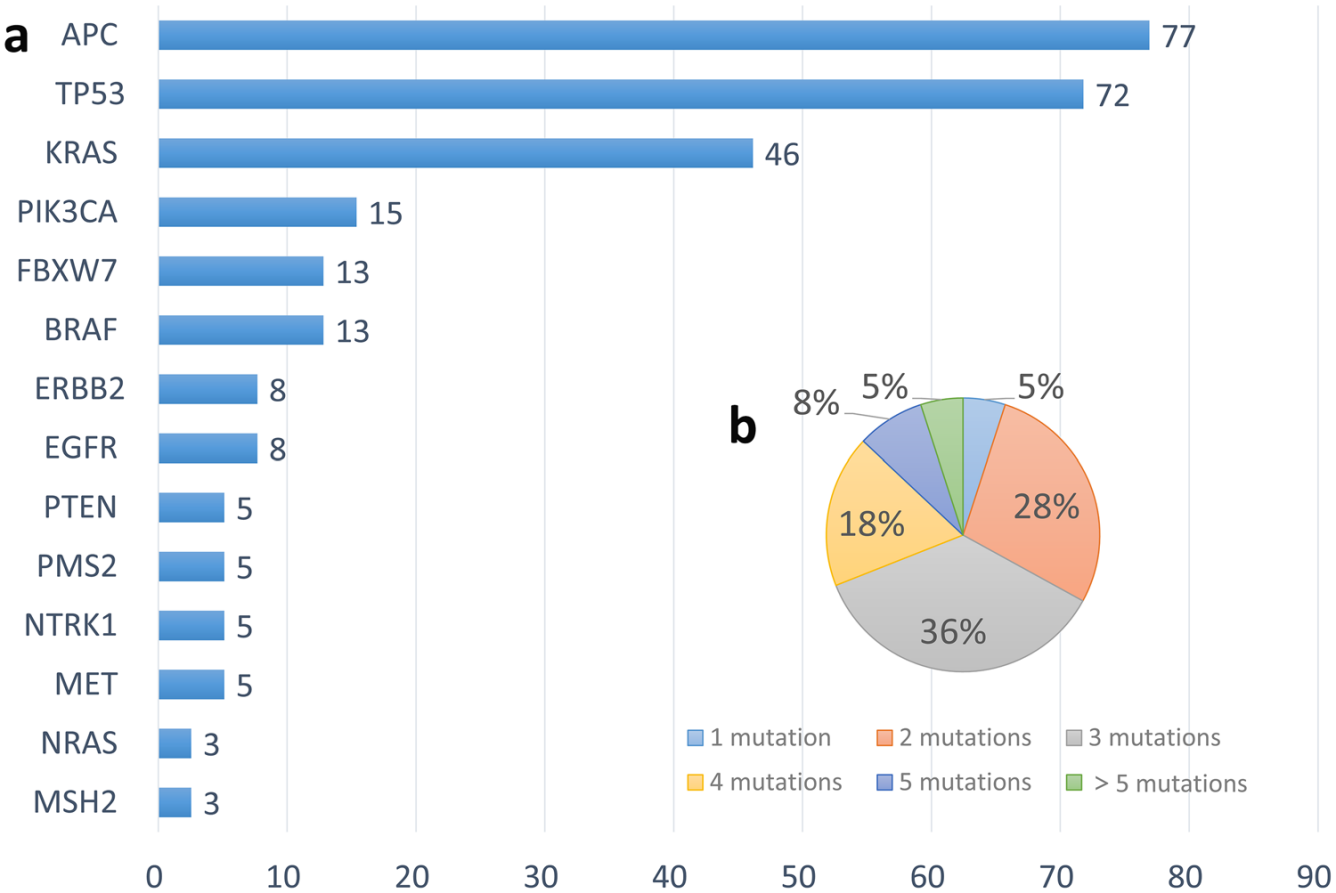
The genetic profile of CRC. **a** Bar chart showing the frequency of gene mutations in 39 CRC patients. **b** Distribution of altered gene numbers in 39 CRC patients

The landscape of driver mutations is shown in Fig. 1. The most frequently mutated genes were APC (30 of 39, 77%), TP53 (28 of 39, 72%), and KRAS (18 of 39, 46%), which have all been reported as well-known CRC driver genes. The second-most frequently mutated genes included PIK3CA (15%), FBXW7 (13%), BRAF (12%), ERBB2 (8%), EGFR (8%), PTEN (5%), PMS2 (5%), NTRK1 (5%), MET (5%), NRAS (3%), and MSH2 (3%). In the 39 samples tested, one or more genomic alterations were identified in all patients (Fig. 1b), 2 (5%) patients displayed only one mutation, 11 (28%) displayed two mutations, 14 (36%) showed three mutations, 7 (18%) had four mutations, 3 (8%) displayed five mutations, and 2 (5%) displayed more than 5 mutations.

In this study, a total of 27 alterations in the *APC* gene were detected by targeted Next Generation Sequencing (NGS) in 77% (30/39) of all cases, most of which were nonsense mutations (Y935*, Q1291*, R499*, W1049*, E1309*, E1306*, E1306*, Q1429*, E287*, Q1367*, E1309*, L698*, E1169*, R876*, K1370*). Twelve variants have been identified as frameshift mutations (G524fs, T1556fs, T935fs, D1318fs, L1488fs, V1099fs, I1401fs, I1401fs, T1332fs, L1382fs, T1556fs, L1489fs) and three were missense mutations (G2227V, G2227V, F1396L). *TP53* and *KRAS* mutations were observed in 72% (28/39) and 46% (18/39) of the cases, respectively. Six patients (13%) had *PIK3CA* mutations, half of the patients had E545K mutations and the remainder showed H419P, H1047R, and I112F mutations, as depicted in Table 2.

**Table 2:** Mutations detected in CRC samples

Genes	n (Frequency)	Mutation
*APC*	77%(30/39)	Y935*, Q1291*, R499*, W1049*, E1309*, E1306*, E1306*, Q1429*, E287*, Q1367*, E1309*, L698*, E1169*, R876*, K1370*, G524fs, T1556fs, T935fs, D1318fs, L1488fs, V1099fs, I1401fs, I1401fs, T1332fs, L1382fs, T1556fs, L1489fs, G2227V, G2227V, F1396L
*TP53*	72%(28/39)	V157F, Y103fs, Y163C, R273H, A161T, Y220C, G108fs, R248Q, M237I, V157R, V157R, R282W, P152L, P152fs, G245S, R281H, G266V, R342*, R248W, R213*, C135F
*KRAS*	46%(18/39)	G12V, G12D, T58T, G12S, A146T, Q61H, G13D, A59T
*PIK3CA*	13%(6/39)	E545K, H419P, H1047R, I112F

### The CRC patient microbiome differs significantly from that of healthy controls

**Fig. 2:**
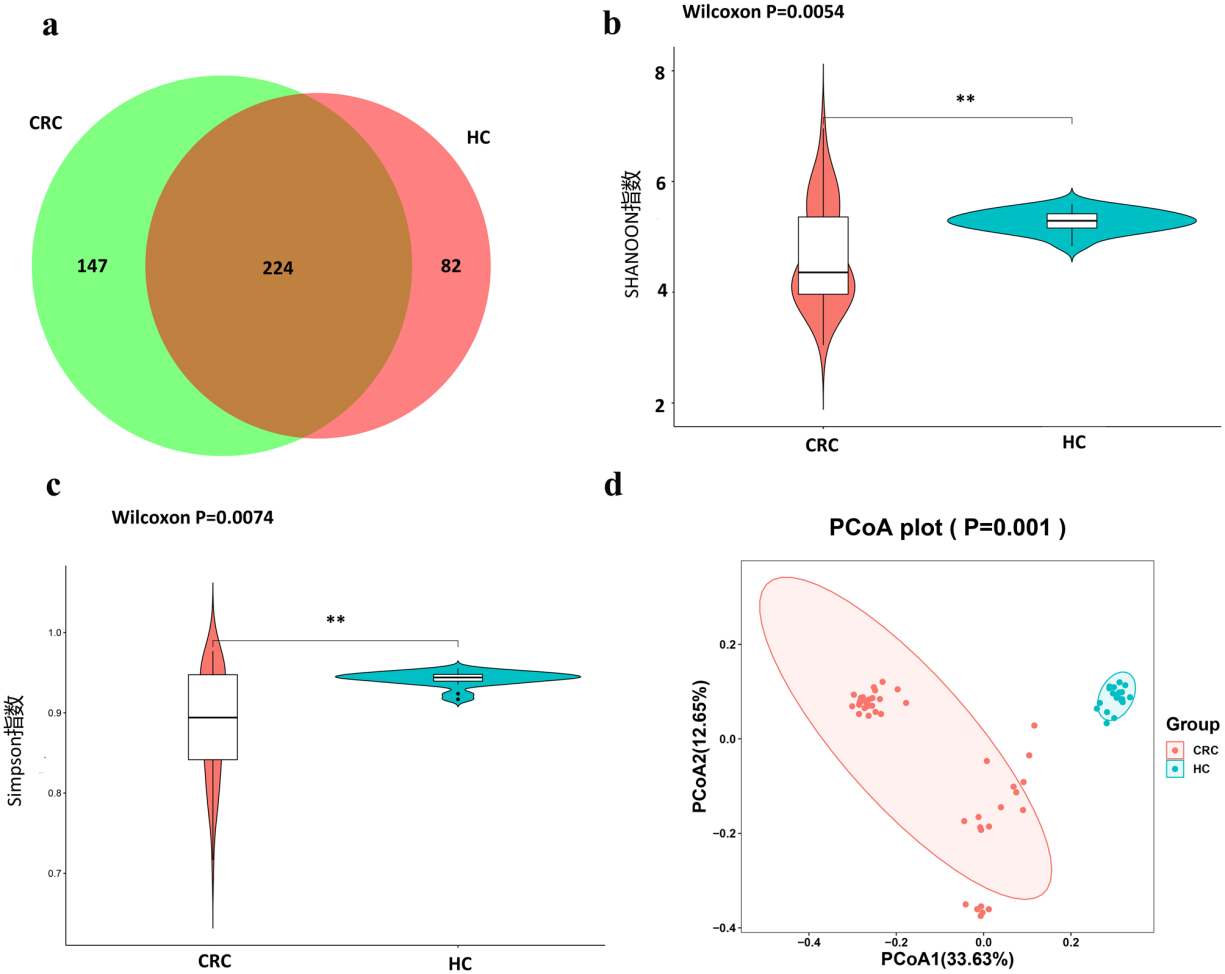
Alterations of fecal bacterial microbiota profile. **a** Venn diagram intuitively presents the number of the common and exclusive OTUs between the CRC and the HC group calculated through R software. **b** The boxplot of Shannon index shows the difference in OTU diversity between the CRC and the HC group (p = 0.0054). **c** The boxplot of Simpson index shows the difference in OTU diversity between the CRC and the HC group (p = 0.0074). **d** PCoA using Bray–Curtis of beta diversity in CRC and HC groups. CRC, colorectal cancer; HC, healthy controls

The number of OTUs obtained in the study was 453, with 371 OTUs belonging to the CRC sample group and 306 OTUs belonging to the HC sample group. 224 OTUs were shared between the CRC and HC group as shown in the Venn diagram (Fig. 2a). The significantly higher alpha diversity indices in the HC group showed that the diversity of the bacterial communities in the CRC group were apparently lower than that of the control group. Moreover, the Shannon index of the HC group was significantly higher than that of the CRC group (p = 0.0054) as well as the Simpson index (p = 0.0074) (Fig. 2b, c). In addition, the principal co-ordinates analysis (PCoA) plot showed differences in the gut microbiota composition. The separated dots suggest that the composition of the microbial structure between samples was more dissimilar (Fig. 2d). Thus, the microbial composition of CRC patients differs significantly from that of healthy control subjects.

### Bacterial composition at the phylum and genus level

**Fig. 3:**
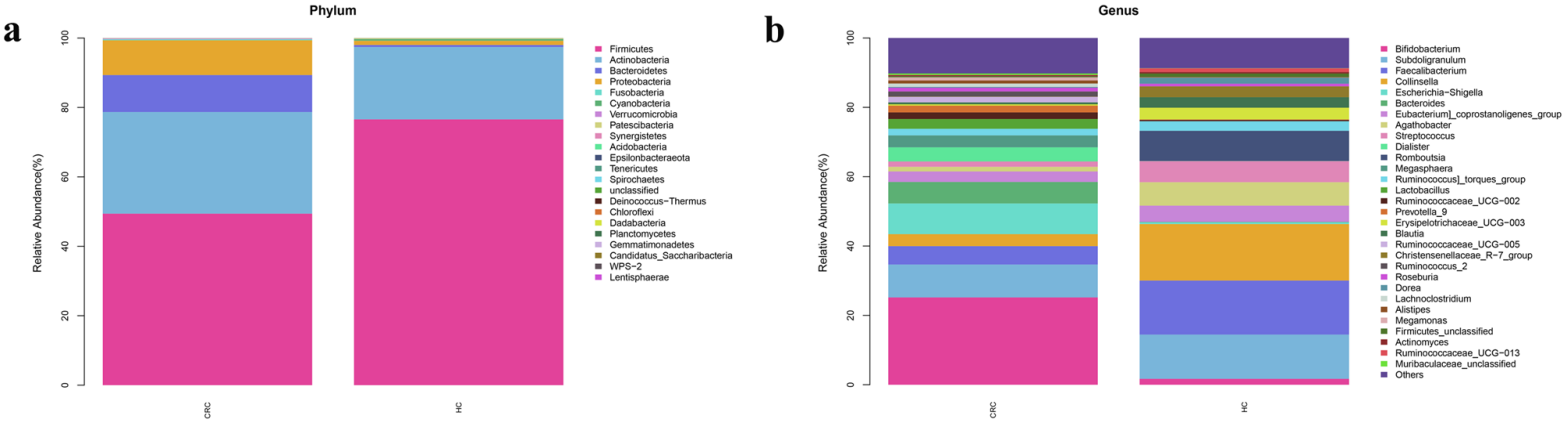
The taxonomic classification of bacterial communities from feces in the CRC and the HC group at level of the phylum (**a**) and genus (**b**)

A histogram of the relative microbiome organism abundance can be used to identify groups and individuals with a higher relative abundance at different classification levels. The structure of the intestinal flora at the phylum and genus level is shown in Figs. 3a, b. At the phylum level, Firmicutes and Actinobacteria were the dominant flora of the two groups and the abundance of Bacteroidetes and Proteobacteria in the HC group was lower than that of the CRC group. At the genus level, *Bifidobacterium* and *Subdoligranulum* were the major flora components in the CRC group and *Faecalibacterium*, *Subdoligranulum* and *Collinsella* in the HC group, respectively. The abundance of *Bifidobacterium*, *Escherichia-Shigella,* and *Bacteroides* in the HC group was lower than that in the CRC group and the abundance of *Collinsella*, *Faecalibacterium*, *Romboutsia*, *Coprobacillus,* and *Streptococcus* in the CRC group was lower than that in the HC group.

**Fig. 4:**
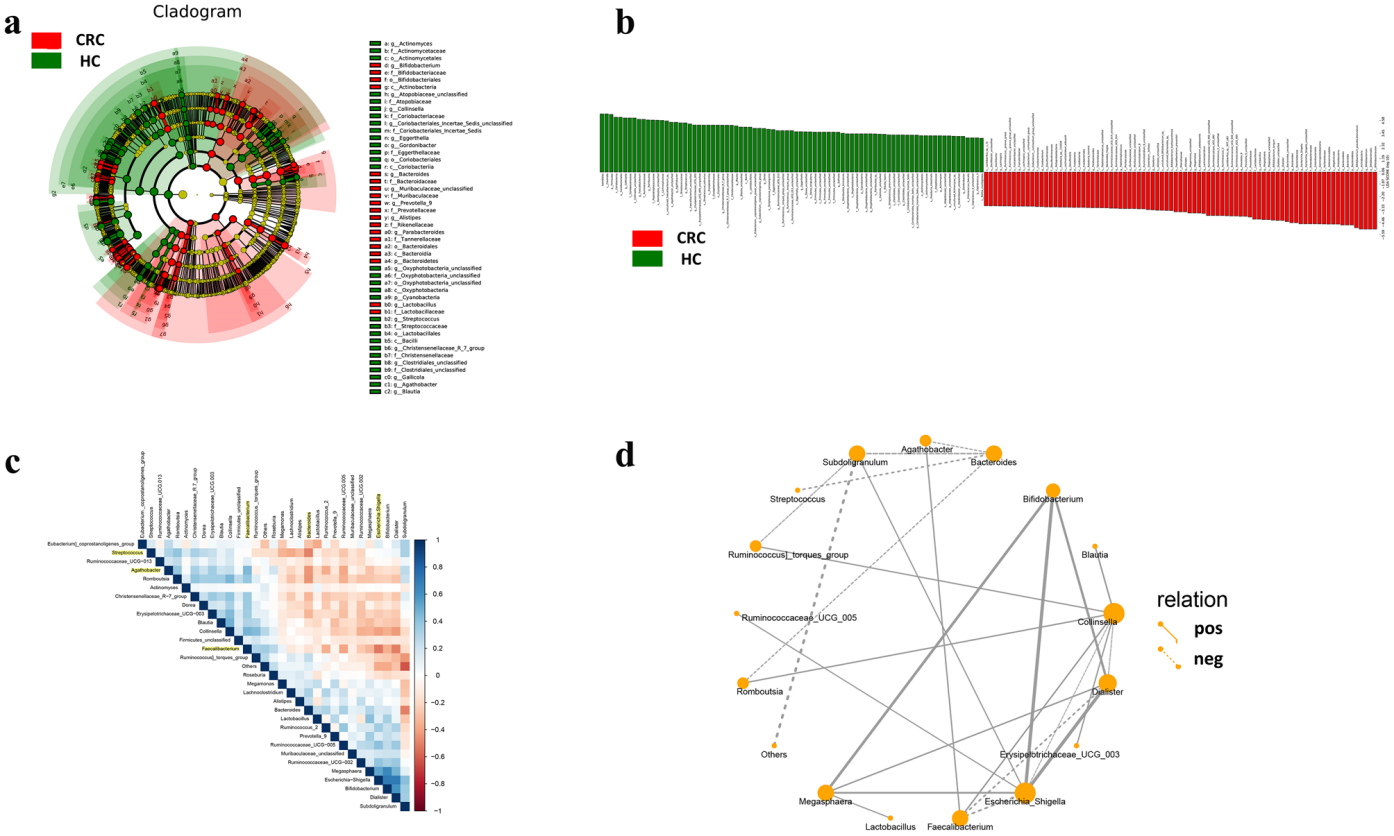
Difference of fecal microbiota in CRC patients and HC. **a** Different circle layers radiate from the inside to the outside to represent the seven classification levels of genus and species of the family phylla and each node represents a species classification at that level. The higher the species abundance, the more nodes are present. Yellow colored nodes indicate species showing no significant difference to the comparison group; red nodes indicate species with significant differences and a higher abundance compared to the reference group; green nodes indicate species with significant differences and a lower abundance compared to the reference group. **b** LDA score computed from features differentially abundant in CRC and HC fecal samples. The criteria for feature selection were LDA score > 4, p < 0.05, Green and red represent the HC group and CRC group, respectively. **c** Spearman correlations on the genus level by calculating the microbial abundance of the Top30. Red dots indicate a negative correlation, blue dots indicate a positive correlation, a cross indicates no significant difference (P > 0.05). **d** Interaction network on the genus level by calculating the microbial abundance of the Top30. Solid lines indicate a positive correlation and dotted lines indicate a negative correlation. The thickness of the line represents the association strength. Each dot represents the relative abundance of the species

In order to find those species with a significantly different abundance in the considered groups, a cladogram representation, performed by LDA effect size (LEfSe) analysis, is shown in Fig. 4a. It shows that *Bifidobacterium*, *Bacteroides*, *Dialister,* and *Megasphaera* were significantly enriched in the CRC group while the abundance of *Collinsella*, *Faecalibacterium*, *Romboutsia* and *Agathobacter* was obviously higher in the HC group. The greatest differences in taxa among the groups are also displayed in Fig. 4b. Different microbial abundances were analyzed by performing Spearman correlations as displayed in Fig. 4c. The results show that *Streptococcus* is negatively correlated with *Bacteroides*, as is *Faecalibacterium* with *Escherichia-Shigella* (red), however, the latter is positively correlated with *Agathobacter* (blue). SparCC networks were established to evaluate the interaction correlation for different genera. The results revealed that *Bifidobacterium* is positively correlated with *Megasphaera*, *Bifidobacterium* is positively correlated with *Escherichia-Shigella*, and *Romboutsia* are exclusive for *Bacteroides*, as depicted in Fig. 4d. We also conducted specificity and sensitivity analyses for sample classification. The total area under the curve (AUC) was 0.88 (*Faecalibacterium*), 0.93 (*Collinsella*) and 0.98 (*Bacteroi*des), respectively.

### Changes of the intestinal flora in patients with different gene mutations

**Fig. 5:**
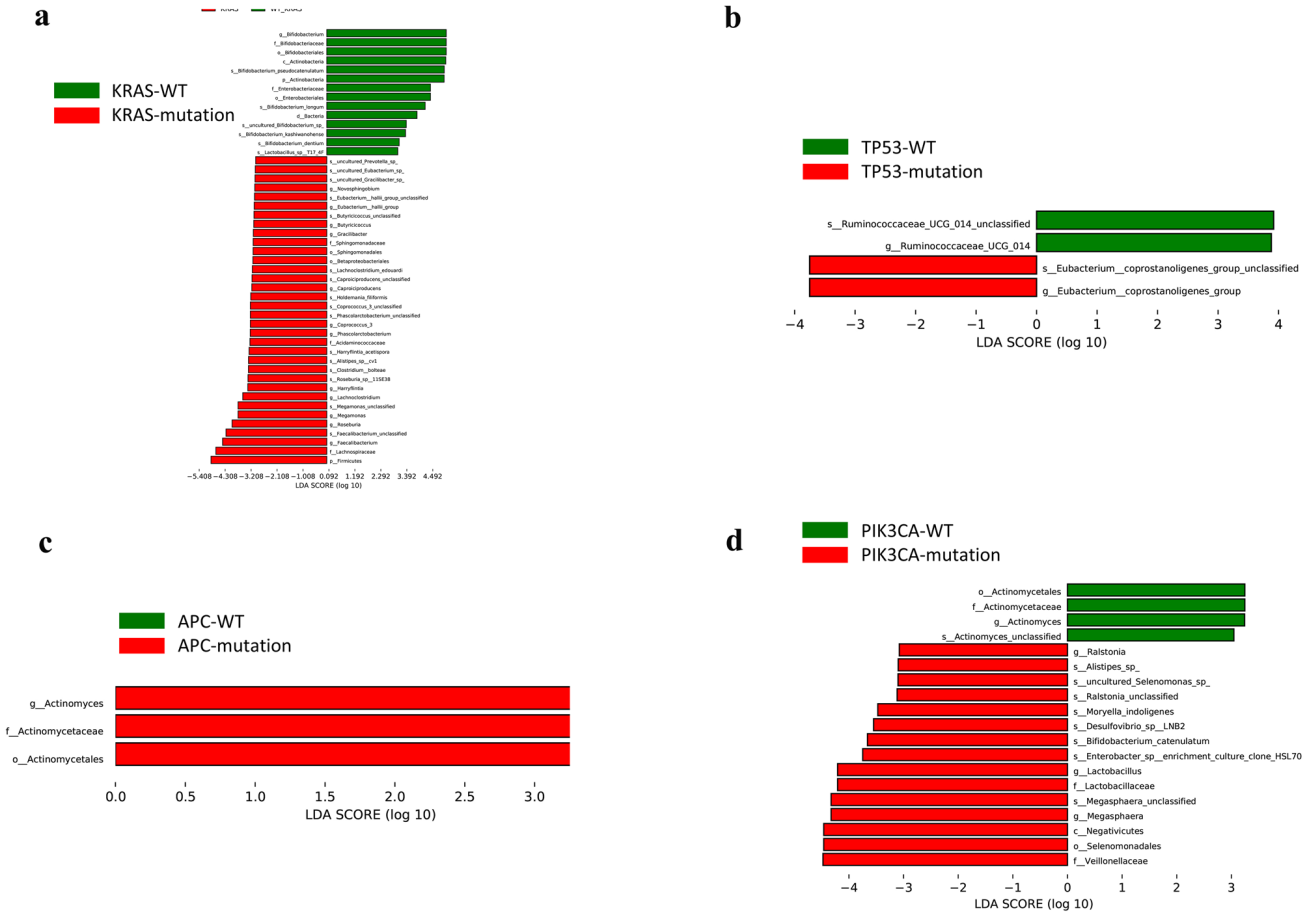
LEfSe was used to compare the microbial variation of the KRAS (**a**), TP53 (b), APC (**c**), and PIK3CA (**d**) groups. The criteria for feature selection were an LDA score > 4 and a p < 0.05

LDA effect size (LEfSe) analysis was performed to identify the specific OTUs that exhibited significantly different abundances between groups in patients with different gene mutations (Fig. 5). Although the number of CRC patients with *KRAS* mutations was not the highest (18/39, Table 2), they carried the most kinds of specific OTUs relative to patients with other mutations and had obviously a higher abundance of *Faecalibacterium*, *Roseburia*, *Megamonas*, *Lachnoclostridium,* and *Harryflintia* and a lower abundance of *Bifidobacterium* at the genus level compared to patients without *KRAS* mutations (Fig. 5a). At the species level, *Roseburia sp 11SE38*, *Clostridium bolteae*, *Harryflintia acetispora*, *Alistipes sp cv1*, et al. were observed to have a higher abundance in CRC patients with *KRAS* mutations while *Bifidobacterium longum*, *Bifidobacterium dentium,* and *Bifidobacterium kashiwanohense* had a higher abundance in patients without this mutation (Fig. 5a). The mutations of *TP53* and *APC* were associated with *Eubacterium_coprostanoligenes* and *Actinomyces*, respectively (Figs. 5b, c). The abundance of *Megasphaera*, *Lactobacillus,* and *Ralstonia* was significantly higher in CRC patients with *PIK3CA* mutations whereas patients without this mutation had notably a higher abundance of *Actinomyces* (Fig. 5d). Several microbial species were observed to have a significantly higher abundance in CRC patients with *PIK3CA* mutations, including *Bifidobacterium catenulatum*, *Desulfovibrio sp LNB2*, *Moryella indoligenes,* and *Alistipes sp* (Fig. 5d). These results indicate that gene mutations in colorectal cancer are associated with changes in the intestinal microflora.

### Correlation analysis of bacterial community appearance and mutation type 

**Fig. 6:**
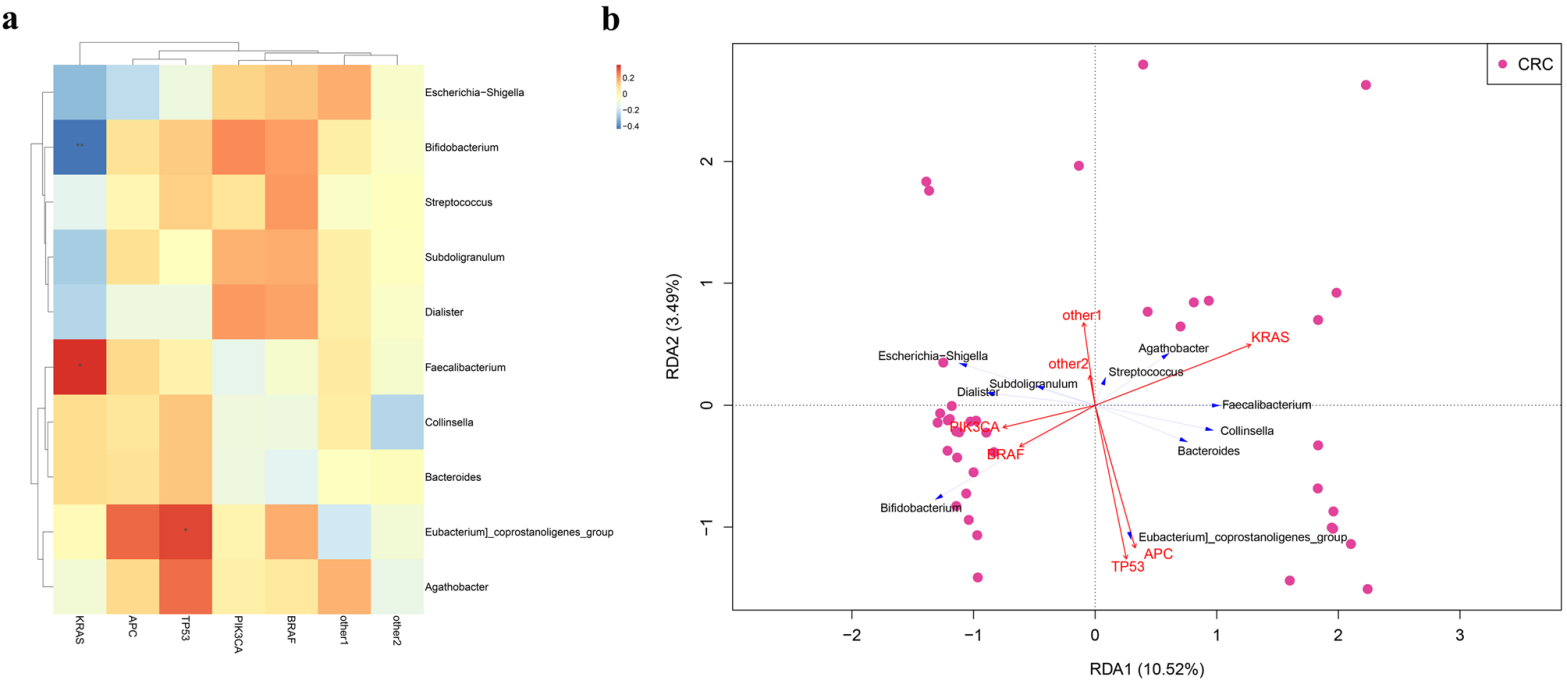
Correlation analysis of bacterial community identity (at the genus level) and mutation type. **a** Spearman correlation heatmap. Red represents a positive correlation and blue represents a negative correlation. **b** Redundancy Analysis (RDA). A blue arrow represents the species, a red arrow represents the mutation type

In order to analyze the correlation between different gene mutation types and the associated relative abundance of microflora components in patients with colorectal cancer and to observe the relationship between different microflora and gene-related clinical indicators, we performed a Spearman correlation as well as a redundancy analysis (RDA). The results of the Spearman correlation analysis showed that the patients with *KRAS* mutations were negatively correlated with *Bifidobacterium* and positively correlated with *Faecalibacterium* while the patients with a *TP53* mutation were positively correlated with *Eubacterium_coprostanoligenes* (Fig. 6a) which is consistent with the results shown in Fig. 4. On the other hand, RDA also indicated a positive correlation between *KRAS* mutations and *Bifidobacterium* as well as *TP53* mutations and *Eubacterium coprostanoligenes* (Fig. 6b). RDA further revealed positive correlations between *ACP* mutations and *Eubacterium coprostanoligenes*, between *BRAF* mutation and *Bifidobacterium*, as well as between *PIK3CA* mutations and *Dialister* (Fig. 6b). Taken together, these results revealed the association of different mutation types with the specific bacteria.

## Discussion

CRC is the third most common malignant tumor in the world and the second most common cause of malignant tumor-related death (about 9.2% of the total number of malignant tumor deaths), ranking first in all gastrointestinal malignancies [13]. Diet, lifestyle, and host genotype may be involved in the occurrence and development of colorectal cancer through metabolic and inflammatory mechanisms [14]. Genes are known to regulate the pathogenesis of CRC and are associated with the survival outcomes of patients. Among these genes are *KRAS*, *TP53*, *APC*, *SMAD4*, *BRAF*, and *PIK3CA* [2]. Gurjao et al. found that overeating of red meat leads to CRC by altering *KRAS* and *PIK3CA* and the alkylation state of these genes [15]. In our study, the driver gene mutations were mainly distributed among *APC*, *TP53*, *KRAS,* and *PIK3CA*. APC inhibits Wnt signaling by promoting phosphorylation and degradation of β-catenin. In intestinal stem cells (ISCs), loss of *APC* function mutations drives intestinal adenomas by enhancing intracellular Wnt signaling [16]. Brandt et al. found that oncogenic *KRAS*, together with β-Catenin, favoured the expansion of crypt cells with high ERK activity [17].

At present, molecular targets of colon cancer comprise EGFR, VEGF, ERBB2, BRAF, KRAS, PD-1, CTLA-4, NTRK etc. The targeted therapy of CRC patients displaying EGFR and EGFR-related pathway gene mutations can be divided into those using monoclonal antibodies and those using small molecule tyrosine kinase inhibitors (TKIs). The used monoclonal antibodies comprise anti-EGFR monoclonal antibodies such as cetuximab and panizumab, anti-HER2 monoclonal antibodies such as pertuzumab and trastuzumab, and the Insulin like growth factor 1 receptor (IGF1R) inhibitor such as darlozumab. TKIs include BRAF inhibitors such as vimofinib, Darafenib, and encofanil, MEK inhibitors comprise trametinib, cobitinib, bemitinib, and Selumetinib, and eventually, the HER2 inhibitor lapatinib (dual EGFR and HER2 targeting), etc. Cetuximab can bind to EGFR on the surface of tumor cells, competitively blocks the EGFR signaling pathway and inhibits the proliferation of tumor cells. Cetuximab is widely used in the treatment of CRC patients with KRAS/NRAS/BRAF wild-type genome. Currently, the treatment of patients with KRAS mutation is the focus of targeted therapy. Sotorasib has been approved for the treatment of NSCLC with KRAS mutations by the FDA. The CodeBreak 101 study showed that the objective response rate (ORR) of Sotorasib combined with panitumumab achieved 27% in patients with advanced/metastatic CRC with a KRAS G12C mutation and the disease control rate (DCR) reached 81%.

Similar to key metabolic and immunomodulatory agents, the intestinal flora is believed to play an important role in the development of colorectal cancer [18]. There is a rich microbiota in the colon lumen. Previous studies have shown that dysbiosis and imbalance in the gut microbiome can mediate or alter the impact of environmental factors on CRC risk [19, 20]. In this study, we depicted the overall composition of the gut microbiota by 16S rRNA sequencing, demonstrating that microbial dysbiosis is characterized by a distinct microbial composition and altered relative abundances of species with specific functions. Compared to healthy controls, the CRC patient group displayed a decreased microbial diversity and an increased microbial richness which is consistent with arguments outlined in previous reports [21–23]. Additionally, our study could show that at the phylum level, *Bacteroidetes* and *Proteobacteria* were enriched and at the genera level, *Bifidobacterium*, *Shigella Escherichia coli* and *Bacteroides* were more abundant in the CRC group. This is in accordance to previous studies that showed a higher abundance of Bacteroidetes and Proteobacteria in the CRC patients [24]. The relationship between *Fusobacteria* and CRC was studied on a large mass cohort comprising 3,157 individuals, including CRC patients and healthy controls, and revealed that *Fusobacterium varium* and *Fusobacterium ulcerans* were related to a homologue of FadA adhesin [25]. Ma et al. reported that colorectal tumor apoptosis is induced by sitosterol through promoting gut microbiota to produce SCFAs which display antiproliferative effects on human colorectal cancer cells via gene expression inhibition [26]. Also, the intestinal flora may promote serrated lesions through EGFR signaling, the induction of cellular proliferation, the activation of a tumor immunosuppressive microenvironment, and the induction of an inflammatory response [27]. ROC analysis suggested that *Faecalibacterium*, *Collinsella,* and *Bacteroides* may be potential biomarkers for CRC.

Host genes can also regulate the growth of microbiota and influence the composition of the intestinal microbial community. Thus, to achieve a comprehensive analysis, we investigated the association between host gene mutations and microbial composition in CRC patients. The most significant associations of host gene mutations with gut bacterial composition were a higher abundance of *Faecalibacterium* and lower abundance of *Bifidobacterium* in patients with *KRAS* mutations, a higher abundance of *Eubacterium coprostanoligenes* in *TP53* mutated patients, and a higher abundance of *Lactobacillus* in *PIK3CA* mutated patients compared to patients without these gene mutations. This suggests that host driver mutations affect the gut microbiota composition in CRC patients. We will explore the relationship between the effect of targeted therapy and the intestinal flora in these patients with gene mutations.

A previous CRC study reported that *Faecalibacterium* was enriched in the survival group whereas *Fusobacterium nucleatum* and *Bacteroides fragilis* were more abundant in the worse prognosis groups which is consistent with our findings, at least to some extent [28]. Specifically, *Faecalibacterium* displays an anti-inflammatory effect and its metabolites block the activation of NF-kB and the secretion of IL-8 [29]. Interestingly, *KRAS* mutations happen to be more common in colorectal serrated adenocarcinoma that involves the NF-kB pathway and the secretion of IL-8 which may account for the association of the higher abundance of *Faecalibacterium* in *KRAS* mutated CRC patients [30, 31]. Moreover, *Lactobacillus* was more abundant in CRC patients with *PIK3CA* mutations in our study. *Lactobacillus* inhibits the growth of colorectal cancer by secreting short-chain fatty acids (SCFAs) that enhance the intestinal barrier function [32]. Taken together, our findings are supported by the above-mentioned reports, which highlight the clinical relevance of *Faecalibacterium* and *Lactobacillus* in the development of CRC and suggest their potential to become a therapeutic agent by producing bioactive compounds that may benefit the host. We hypothesized that this relationship between host driver mutations and intestinal flora might play a pivotal role in the pathogenesis of CRC and provided a new idea for the treatment of CRC in the future. However, further detailed studies are needed to confirm our findings and to investigate the molecular mechanisms of host driver mutations and their effects on gut microbiota in CRC patients.

Although our study indicated an association of the intestinal flora composition with gene mutations such as *KRAS* and *TP53*, our results have some limitations. In this analysis, the number of patients included was not large enough. Besides, only a small panel of 18 genes was tested in this study. We expect that a larger panel of NGS parameters as well as a larger sample size will hopefully lead to more discoveries in the future. Combined analysis of multi-omics data, such as transcriptome and methylome, must be conducted for further experimental studies and clinical trials to validate and reinforce our findings.

In conclusion, we revealed a potential relationship between the host genome and the gut microbe composition in CRC patients. The results of the gene and intestinal flora analyses provided a clearer understanding of the pathogenesis of colorectal cancer. The correlation between relative bacterial abundance and host gene mutations suggests that intestinal microbiota may influence the growth of mutated cells and that their dysregulated cellular pathways influence the abundance of specific bacteria. Thus, our study might provide a new perspective therapeutic approach for the treatment of CRC.

## Data Availability

Data and materials used and/or analyzed during the current study are available from the corresponding author on reasonable request.
